# Effects of Angiotensin Receptor-Neprilysin Inhibitors Versus Enalapril or Valsartan on Patients With Heart Failure: A Systematic Review and Meta-Analysis

**DOI:** 10.7759/cureus.41566

**Published:** 2023-07-08

**Authors:** Arpit Jain, Shourya Meyur, Lovish Wadhwa, Kamaldeep Singh, Rishi Sharma, Ishita Panchal, Giustino Varrassi

**Affiliations:** 1 Emergency Medicine, All India Institute of Medical Sciences, New Delhi, New Delhi, IND; 2 Medicine, AMA School of Medicine, Makati, PHL; 3 Internal Medicine, Sambhunath Pandit Hospital, Kolkata, IND; 4 Medicine, Ramaiah Medical College, Bangalore, IND; 5 Cardiology, Government Medical College & Hospital, Chandigarh, IND; 6 Internal Medicine, Jawaharlal Nehru Medical College, Chandigarh, IND; 7 Medicine, D. Y. Patil Medical College, D. Y. Patil Education Society Deemed University, Kolhapur, IND; 8 Internal Medicine, Jawaharlal Nehru Medical College, Belagavi, IND; 9 Pain Medicine, Paolo Procacci Foundation, Rome, ITA

**Keywords:** systematic review, enalapril, meta-analysis, valsartan, heart failure, sacubitril/valsartan, arni, angiotensin receptor–neprilysin inhibitors

## Abstract

Recent studies have focused on treating heart failure, primarily mitigating symptoms and reducing the risk of mortality and other cardiovascular complications. A promising new treatment approach involves using LCZ696, an angiotensin receptor-neprilysin inhibitor (ARNI) comprising sacubitril and valsartan. This treatment is superior to the conventional drugs enalapril or valsartan in patients diagnosed with heart failure. A systematic search was conducted on PubMed, the Cochrane Library, and Elsevier’s ScienceDirect databases to identify studies comparing sacubitril/valsartan with other drugs in heart failure patients with reduced ejection fraction (HFrEF) and preserved ejection fraction (HFpEF). The analyses were conducted using the random-effects model. The study's primary outcomes included all-cause mortality, death from cardiovascular causes, first hospitalization for heart failure, congestive heart failure, and changes in the Kansas City Cardiomyopathy Questionnaire (KCCQ) clinical score. The pooled analysis showed that treatment with the sacubitril/valsartan combination was associated with a significantly decreased rate of first hospitalization for heart failure (RR: 0.86; 95% CI: 0.79, 0.98, p: 0.03; I2: 57%) and significantly increased KCCQ clinical score (WMD: 2.20; 95% CI: 0.33, 4.06, p: 0.02; I2: 100%). However, the two groups had no significant difference in all-cause mortality (RR: 0.90; 95% CI: 0.80, 1.01, p: 0.08; I2: 20%), death from cardiovascular causes (RR: 0.96; 95% CI: 0.87, 1.05, p: 0.34; I2: 0%), or congestive heart failure (RR: 0.97; 95% CI: 0.75, 1.25, p: 0.19; I2: 38%). The research findings suggest that sacubitril/valsartan (LCZ696) reduces hospitalizations due to heart failure and improves KCCQ clinical scores. This treatment also reduces the decline in renal function and side effects associated with enalapril or valsartan. Nonetheless, further high-quality randomized controlled trials with large sample sizes are needed to assess other impacts of this therapy on heart failure patients. Overall, the use of LCZ696 represents a promising new approach to the treatment of heart failure.

## Introduction and background

Heart failure (HF) is historically defined as a disease characterized by an impaired heart capacity to pump or refill blood effectively [1]. As an alternative, it may originate from a defect in the anatomy or physiology of the heart, leading to inadequate cardiac output or, in certain circumstances, adequate cardiac output but with the anticipatory neurohormonal response and elevated pressure in the left ventricle. Overall, HF is a complex ailment that, if neglected, can have a negative impact on a person's health [1]. The 2019 Heart Failure Association (HFA) ATLAS provided projections regarding essential aspects of HF epidemiology throughout Europe and pointed out that the prevalence of HF differs significantly, varying from less than 12 per 1,000 people in Spain and Greece to more than that [2]. Thirty per 1,000 people in Lithuania and Germany. In 2012, 2.4% was the reported statistic for HF in the United States. The comparable estimates of prevalence in Asia vary between 1.3% and 6.7% [2].

The European Society of Cardiology's medical guidelines have recently been amended. They currently recommend (Class I) the use of LCZ696, an angiotensin receptor-neprilysin inhibitor (ARNI), to minimize mortality as well as morbidity among individuals with HF with reduced ejection fraction (HFrEF) [2,3]. LCZ696 is a composite of sacubitril and valsartan, possessing the molecular components of both drugs [3]. The combined medication of sacubitril/valsartan has the ability to hinder the activity of both neprilysin and angiotensin receptors. This presents the advantage of concomitantly obstructing pro-fibrotic and hypertrophic mechanisms (components of angiotensin receptor blockers (ARBs)) while also promoting anti-fibrotic and anti-hypertrophic mechanisms (components of neprilysin inhibition (NEPI)) [4]. NEPI increases circulating natriuretic peptide, activates the reflex renin-angiotensin system (RAS), and slows angiotensin II breakdown. These activities may hinder the desired therapeutic outcomes. Thus, NEPI requires RAS suppression. ARBs are preferred over ACEIs and NEPI due to the likelihood of angioedema [5]. Angiotensin-converting enzyme inhibitors (ACEIs) and ARBs are the main treatments for HFrEF. The PARADIGM-HF study found that sacubitril/valsartan outperformed enalapril in lowering the risk of cardiovascular (CV) death and hospitalization for HF [6]. Given these advantages, individuals with HFrEF who have previously used ACEIs, or ARBs are advised to try ARNI therapy as a possible substitute [5,6].

This new drug is intended to replace standard therapy of ACEIs or ARBs for patients with HFrEF and HF with preserved ejection fraction (HFpEF) who continue to experience symptoms even though acquiring the best care possible, including beta-blockers, ACEIs, and mineralocorticoid antagonists [5,6]. This medication is currently being evaluated in its early phases in several patient populations. Our primary objective with this meta-analysis is to compare the safety and effectiveness of sacubitril/valsartan with a control group comprising either enalapril or valsartan in patients diagnosed with HFrEF and HFpEF. Our meta-analysis is unique because it is the first recently available study to conduct a sub-group analysis, which briefly compares ARNI's benefits and side effects with enalapril or valsartan.

## Review

Methods

This meta-analysis adheres to the prescribed guidelines outlined by the Preferred Reporting Items for Systematic Review and Meta-analysis [7].

Data Sources and Search Strategy

Clinical studies were electronically searched in PubMed, the Cochrane Library, and Elsevier's ScienceDirect databases, with the search conducted in March 2023. No language restrictions were applied. To gather relevant literature, a combination of specific keywords and medical subject headings (MeSH) terms such as “heart failure,” “heart failure, systolic,” “heart failure, diastolic,” “dyspnea, paroxysmal,” “edema, cardiac,” “cardio-renal syndrome,” “LCZ696," “LBQ657” (an active metabolite of sacubitril), “neprilysin inhibitor,” “Entresto,” or “valsartan and sacubitril” were used. Adjustments were made to the population, intervention, comparison, and outcome (PICO) methodology. The study population included patients with chronic HF (CHF). Three researchers (AJ, SM, and LW) independently reviewed the titles and abstracts of potentially eligible studies.

Inclusion and Exclusion Criteria

Inclusion criteria: The primary objective of this systematic review was to examine the effectiveness of sacubitril/valsartan compared to a placebo or a reference compound in treating patients with HF. The study established the following inclusion criteria to ensure a comprehensive and dependable analysis. Firstly, only double-blind, randomized controlled trials (RCTs) were considered, as they provide a robust methodological framework for assessing treatment outcomes. Secondly, the intervention of interest involved sacubitril/valsartan compared to either a placebo or a specific reference compound. This comparison allowed for a precise evaluation of the particular effects attributed to sacubitril/valsartan. Thirdly, the study included participants aged 18 and above, as this age range typically represents the HF population. Fourthly, the participants had to receive HF treatment to ensure the findings' relevance to the target population. Finally, the inclusion criteria specified that only articles published in English would be included in the review, ensuring accessibility and consistency in data extraction and analysis. These carefully defined inclusion criteria aimed to facilitate a rigorous and focused analysis of the available literature, providing reliable evidence regarding the efficacy of sacubitril/valsartan in treating HF.

Exclusion criteria: To maintain the quality and dependability of the systematic review, specific exclusion criteria were implemented to filter out studies that did not meet the predetermined standards. The following exclusion criteria were established. Nonclinical studies were excluded from the analysis to maintain the focus on studies involving human subjects, as the review aimed to evaluate the effectiveness of the intervention in a clinical context. Studies lacking control groups were excluded to ensure the availability of appropriate comparison groups, which is crucial for drawing valid conclusions regarding the intervention's efficacy. Observational studies, including cohort studies, case-control studies, cross-sectional studies, case reports, case series studies, as well as editorials, review articles, and conference abstracts, were excluded from prioritizing the inclusion of RCTs, which provide the highest level of evidence for assessing treatment efficacy. Studies with a sample size of less than 20 were excluded to ensure sufficient participants for meaningful statistical analysis and minimize the potential bias associated with smaller sample sizes. Studies with inconclusive or ambiguous results were excluded to focus on those that offered transparent and interpretable findings, thereby enhancing the reliability and validity of the review. Studies for which the full text was unavailable were excluded to ensure the reviewers had access to all the necessary information for a comprehensive analysis. These exclusion criteria were carefully established to maintain methodological rigor and improve the overall quality of the systematic review by selecting studies that met the predefined standards of relevance and reliability.

Data Extraction and Definitions

A standardized data collection form was utilized to extract relevant information from each study, encompassing details such as the author's name, publication year, country, study population, participants' demographic data, medications taken prior to trial enrollment, New York Heart Association (NYHA) functional classification, and clinical outcomes. The primary outcomes encompassed all-cause mortality, death from CV causes, initial hospitalization for HF, congestive HF, and changes in the clinical score of the Kansas City Cardiomyopathy Questionnaire (KCCQ). Secondary outcomes comprised worsening renal function, hyperkalemia, symptomatic hypotension, angioedema, and any treatment-emergent adverse events (TEAEs).

The NYHA Classification provides a straightforward method of classifying the severity of HF based on the patients' physical activity limitations. It categorizes individuals into one of four classes, determined by the extent of their symptoms and restrictions during physical exertion. Class I denotes no symptoms or physical activity limitations, while Class II indicates mild symptoms (such as slight shortness of breath or angina) and minor activity restrictions. Class III represents significant limitations in physical activity due to symptoms, even during less-than-usual exertion, with patients experiencing discomfort when walking short distances (20-100 meters) and finding relief only when at rest. Class IV signifies extreme restrictions, with symptoms persisting even during periods of rest, resulting in patients being primarily confined to bed.

The KCCQ Clinical Score integrates Physical Limitation, Symptom Frequency, Quality of Life, and Social Limitation. At least one of the four scale scores must be present to calculate the summary score. All KCCQ scores are standardized on a scale from 0 to 100 and often summarized in 25-point intervals. These scores reflect various aspects of health status: Very poor to poor scores range from 0 to 24, poor to fair scores range from 25 to 49, fair to good scores range from 50 to 74, and reasonable to excellent scores range from 75 to 100.

Quality Assessment of the Included Studies

All included RCTs were evaluated for quality using the Cochrane risk of bias tool [8].

Data Synthesis

The statistical analysis for this meta-analysis was performed using Review Manager version 5.4.1, a tool developed in collaboration between The Nordic Cochrane Centre and The Cochrane Collaboration in Denmark in 2014. Only comparative studies were included in the analysis. The results were presented using forest plots, illustrating the pooled effect of relative risks (RRs) for dichotomous outcomes and weighted mean differences (WMDs) for continuous outcomes. To ensure the accuracy of the findings, a random-effects model with generic-inverse variance was employed.

A significance level of p < 0.05 was established to determine the significance of the results. Funnel plots were generated for each primary outcome to assess the potential for publication bias. Higgin's I2 test was used to evaluate the level of heterogeneity, categorized as low, moderate, or high. In cases where substantial heterogeneity (> 75%) was observed [9], a sensitivity analysis was conducted by omitting one study at a time to evaluate the influence of individual studies on the overall findings.

All analyses were considered statistically significant if the p-value was less than 0.05. The authors conducted a comprehensive analysis of the data to ensure the accuracy and reliability of their findings. Given that the data utilized in this study were collected and compiled from previous clinical trials where participants had already provided informed consent, seeking approval from an ethics committee for this particular study was unnecessary.

Results

Eligible Studies

**Figure 1:**
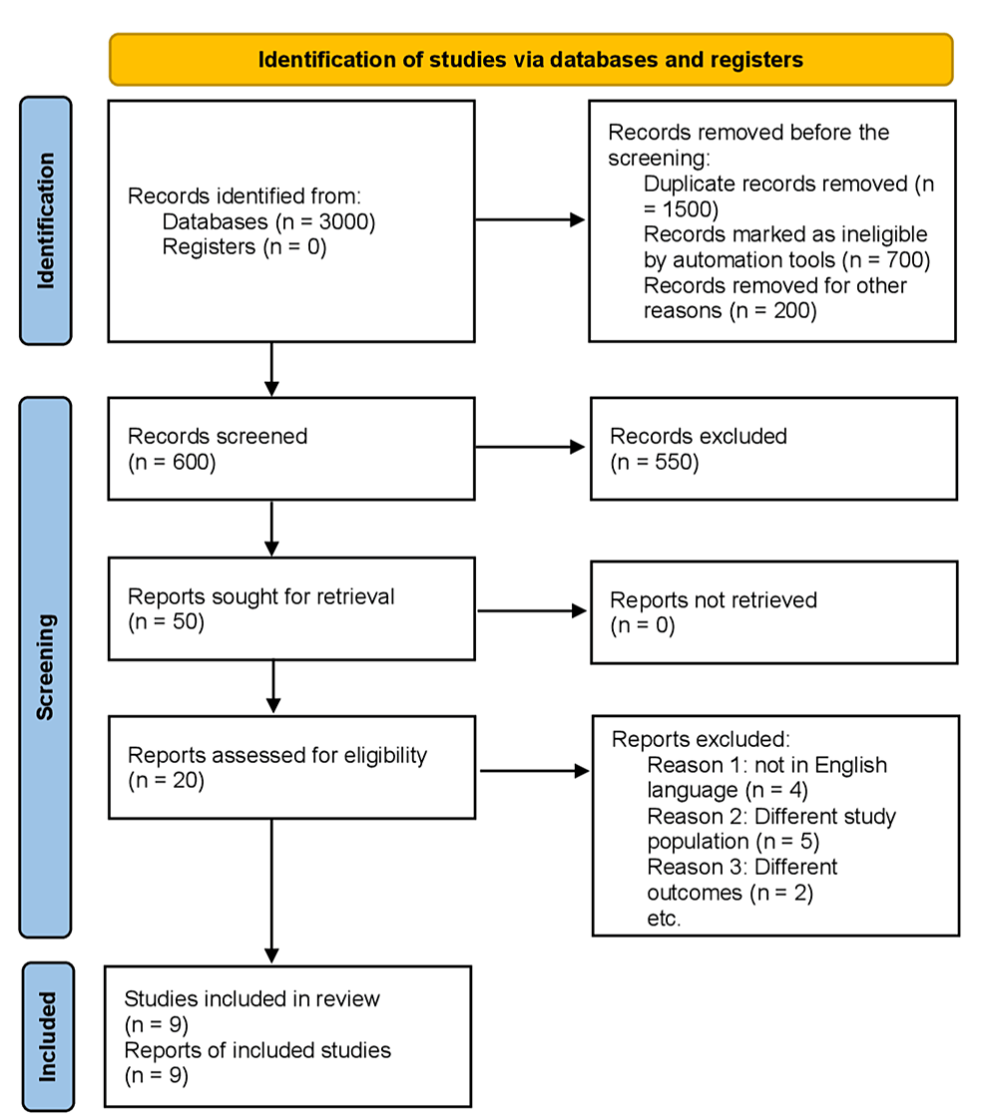
Preferred reporting items for systematic reviews and meta-analyses (PRISMA). PRISMA flow diagram illustrating the search strategy and study selection process for the meta-analysis. The initial search yielded 1,500 articles, which were subsequently screened for duplicates and evaluated based on title and abstract. Nine randomized controlled trials (RCTs) met the inclusion criteria for the analysis, with only comparative studies being included.

Initially, the literature review yielded a total of 3000 articles. By eliminating duplicates and evaluating titles and abstracts, nine RCTs [10-18] were identified and included in this meta-analysis. Figure 1 provides a comprehensive overview of the search strategy employed, depicted in the PRISMA diagram. All nine RCTs were conducted as comparative, multicenter, double-blind studies. Within these studies, three [13-15] utilized Valsartan in the control arm, while the remaining six employed Enalapril. Additionally, two studies [13,14] focused on HF patients with preserved ejection fraction, while the remaining seven included patients with reduced ejection fraction. The mean duration of follow-up across the studies was 13.43 months. The articles included in this meta-analysis span the years 2012 to 2022.

Baseline Characteristics of the Included Patients

This meta-analysis encompassed 16,637 participants, with 8,299 individuals (49.88%) assigned to the ARNI group and 8,338 individuals (50.11%) assigned to the control group. Most of the patients were male, as 11,355 individuals (68.25%) out of 16,637 were male. The mean age of the included patients was 68.8 ± 9.0 years. While many patients were overweight or obese, a few fell within the normal BMI range. Among the participants, 11,114 individuals (66.8%) were categorized as NHYA functional class II, while 4,398 individuals (26.4%) were classified as functional class III. At baseline, most patients had comorbidities such as diabetes, hypertension, or a previous myocardial infarction and were undergoing treatment with beta-blockers, mineralocorticoid antagonists, or other medications. Detailed baseline characteristics of the included patients are presented in Tables 1-4.

**Table 1:** Baseline characteristics of included studies. SD: Standard deviation, BMI: Body mass index, ARNI: Angiotensin receptor-neprilysin inhibitor, HFrEF: Heart failure with reduced ejection fraction, HFpEF: Heart failure with preserved ejection fraction, RCT: Randomized controlled trial, N/A: Not available.

Study and Year	Type of Study	Intervention name	Control name	Population	Follow-up (months)	Total No. of participants	No. of patients		Age (mean ±SD)		Male No. (%)		BMI (mean ±SD)		White No. (%)		Black No. (%)	
							ARNI	control	ARNI	control	ARNI	control	ARNI	control	ARNI	control	ARNI	control
McMurray (2014) [10]	Multicenter Double blind RCT	Sacubitril/ Valsartan	Enalapril	HFrEF	27	8442	4187	4212	63.8±11.5	63.8± 11.3	3308 (79)	3259 (77.3)	28.1±5.5	28.2± 5.5	2763 (66.0)	2781 (66.0)	213 (5.1)	215 (5.1)
Velazquez (2019) [11]	Multicenter Double blind RCT	Sacubitril/ Valsartan	Enalapril	HFrEF	2	881	440	441	61± 4.93	63± 4.44	327 (74.3)	308 (69.8)	30.5±2.76	30± 2.59	261 (59.3)	254 (57.6)	158 (35.9)	158 (35.8)
Desai (2019) [12]	Multicenter Double blind RCT	Sacubitril/ Valsartan	Enalapril	HFrEF	3	464	231	233	67.8±9.8	66.7± 8.5	170 (74)	185 (79)	30± 5.7	30.1± 5.8	166 (72)	175 (75)	62 (27)	53 (23)
Solomon (2019) [13]	Multicenter Double blind RCT	Sacubitril/ Valsartan	Valsartan	HFpEF	35	8442	4187	4212	72.7±8.3	72.8± 8.5	1166 (48.4)	1151 (48.17)	30.2±4.9	30.3± 5.1	1963 (81.6)	1944 (81.4)	52 (2.2)	50 (2.1)
Solomon (2012) [14]	Multicenter Double blind RCT	Sacubitril/ Valsartan	Valsartan	HFpEF	8	301	149	152	70.9±9.4	71.2± 8.9	64 (43)	67 (44)	30.1±5·5	29.8± 6·1	N/A	N/A	N/A	N/A
Douglas (2022) [15]	Multicenter Double blind RCT	Sacubitril/ Valsartan	Valsartan	HFrEF	6	335	167	168	60.2 13.4	58.3± 13.1	120 (72)	125 (74)	29.5 ±7.5	30.3± 7.8	98 (59)	103 (61)	64 (38)	63 (38)
Piepoli (2021) [16]	Multicenter Double blind RCT	Sacubitril/ Valsartan	Enalapril	HFrEF	4	619	309	310	67.1 11	66.6± 10.4	238 (77)	249 (80.3)	29.3±4.72	29.3± 4.67	N/A	N/A	N/A	N/A
Hiroyuki (2021) [17]	Multicenter Double blind RCT	Sacubitril/ Valsartan	Enalapril	HFrEF	33.9	223	111	112	69 9.7	66.7± 10.9	96 (86.5)	96 (85.7)	23.8±4.0	25.1± 4.2	N/A	N/A	N/A	N/A
Ambrosy (2020) [18]	Multicenter Double blind RCT	Sacubitril/ Valsartan	Enalapril	HFrEF	2	576	298	278	63± 4.19	66± 4.44	226 (75.8)	200 (71.9)	31± 2.71	30± 2.46	N/A	N/A	116 (39)	117 (42)

**Table 2:** Baseline cardiovascular parameters. SD: Standard deviation, ARNI: Angiotensin receptor-neprilysin inhibitor, SBP: Systolic blood pressure, DBP: Diastolic blood pressure, LVEF: Left ventricular ejection fraction, NT-pro-BNP pg/mL: N-terminal pro–B-type natriuretic peptide in PICO gram/milliliter, NYHA: New York Heart Association, N/A: Not available.

Study and Year	Heart rate, beats/min (mean±SD)		SBP (mean±SD)		DBP (mean±SD)		Ischemic cardiomyopathy No. (%)		LVEF % (mean± SD)		Median NT-pro-BNP pg/ml (mean± SD)		NYHA functional class — No. (%)									
													Class I		Class II		Class III		Class IV		Missing	
	ARNI	control	ARNI	control	ARNI	control	ARNI	control	ARNI	control	ARNI	control	ARNI	control	ARNI	control	ARNI	control	ARNI	control	ARNI	control
McMurray (2014) [10]	72± 12	73± 12	122± 15	121± 15	N/A	N/A	2506 (59.9)	2530 (60.1)	29.6± 6.1	29.4± 6.3	1631± 560	1594± 597	180 (4.3)	209 (5.0)	2998 (71.6)	2921 (69.3)	969 (23.1)	1049 (24.9)	33 (0.8)	27 (0.6)	7 (0.2)	6 (0.1)
Velazquez (2019) [11]	81± 5	80± 5	118± 5.67	118± 5.67	N/A	N/A	N/A	N/A	24± 2.96	25± 2.46	2883± 936	2536± 877	4 (0.9)	5 (1.1)	100 (22.7)	122 (27.7)	283 (64.3)	269 (61.0)	39 (8.9)	36 (8.2)	14 (3.2)	9 (2.0)
Desai (2019) [12]	68± 11	68± 12	131± 15	130± 13	77± 10	78± 10	137 (59)	146 (63)	34± 10	33± 10	560± 307	595± 294	33 (14)	28 (12)	152 (66)	161 (69)	56 (20)	44 (19)	N/A	N/A	N/A	N/A
Solomon (2019) [13]	70.6± 12.3	70.3± 12.2	130.5± 15.6	130.6± 15.3	N/A	N/A	899 (37.4)	824 (34.5)	57.6± 7.8	57.5± 8.0	904± 276	915± 289	73 (3.0)	64 (2.7)	1866 (77.5)	1840 (77.0)	458 (19.0)	474 (19.8)	8 (0.3)	11 (0.5)	2 (0.1)	0
Solomon (2012) [14]	69± 12	69± 12	136± 3.7	136± 4.6	80± 2.7	78± 3.45	N/A	N/A	58± 7·3	58± 8·1	828± 217	939± 224	1 (1)	1 (1)	120 (81)	119 (78)	28 (19)	32 (21)	N/A	N/A	N/A	N/A
Douglas (2022) [15]	81.4± 15.0	81± 14.9	113.4± 13.6	112.4± 16.8	N/A	N/A	140 (84)	121 (72)	19.9± 6.2	20.9± 6.8	3449.6± 6616.2	2779.4± 3115.2	3 (2)	5 (3)	38 (23)	37 (22)	67 (40)	70 (42)	59 (35)	55 (33)	0	0
Piepoli (2021) [16]	70.81± 12.91	70.22± 11.59	126.3± 16.45	126.0± 15.84	76.09 (10.13)	76.02 (10.42)	177 (57.28)	174 (56.13)	N/A	N/A	N/A	N/A	N/A	N/A	161 (52.10)	162 (52.26)	146 (47.25)	146 (47.10)	2 (0.65)	2 (0.65)	N/A	N/A
Hiroyuki (2021) [17]	73.9± 13.8	72.3± 12.2	123.6± 17.8	121.2± 14.4	72.8 (13.2)	72.8 (11.9)	N/A	N/A	28.6± 5.1	27.7± 5.5	837± 225.4	841± 269	4 (3.6)	4 (3.6)	101 (91.0)	104 (92.9)	6 (5.4)	4 (3.6)	0	0	0	0
Ambrosy (2020) [18]	80± 4.69	78± 4.44	118± 4.19	119± 4.19	N/A	N/A	N/A	N/A	25± 2.96	25± 2.46	3010± 1046	2731± 989.6	5 (2)	3 (1)	57 (24)	55 (24)	154 (63)	143 (63)	27 (11)	27 (12)	55 (18.4)	50 (17.9)

**Table 3:** Co-morbidities of the patients in the included studies. ARNI: Angiotensin receptor-neprilysin inhibitor, N/A: Not available

Study and Year	Hypertension No. (%)		Diabetes No. (%)		Atrial fibrillation No. (%)		Hospitalization for heart failure No. (%)		Myocardial infarction No. (%)		Stroke No. (%)	
	ARNI	control	ARNI	control	ARNI	Control	ARNI	control	ARNI	control	ARNI	control
McMurray (2014) [10]	2969 (70.9)	2971 (70.5)	1451 (34.7)	1456 (34.6)	1517 (36.2)	1574 (37.4)	2607 (62.3)	2667 (63.3)	1818 (43.4)	1816 (43.1)	355 (8.5)	370 (8.8)
Velazquez (2019) [11]	N/A	N/A	N/A	N/A	N/A	N/A	N/A	N/A	N/A	N/A	N/A	N/A
Desai (2019) [12]	N/A	N/A	N/A	N/A	N/A	N/A	128 (55)	115 (49)	N/A	N/A	N/A	N/A
Solomon (2019) [13]	2304 (95.7)	2280 (95.4)	1046 (43.5)	1016 (42.5)	775 (32.2)	777 (32.5)	1135 (47.2)	1171 (49.0)	561 (23.3)	522 (21.9)	266 (11.1)	242 (10.1)
Solomon (2012) [14]	142 (95)	140 (92)	61 (41)	53 (35)	60 (40)	65 (43)	59 (40)	68 (45)	32 (21)	30 (20)	N/A	N/A
Douglas (2022) [15]	118 (71)	115 (69)	74 (44)	83 (49)	72 (43)	80 (48)	N/A	N/A	61 (37)	60 (36)	22 (13)	27 (16)
Piepoli (2021) [16]	213 (68.93)	203 (65.48)	96 (31)	117 (37.7)	147 (47.57)	147 (47.57)	N/A	N/A	137 (44.34)	145 (46.77)	22 (7.12)	25 (8.06)
Hiroyuki (2021) [17]	71 (64.0)	82 (73.2)	52 (46.8)	52 (46.4)	36 (32.4)	40 (35.7)	80 (72.1)	82 (73.2)	51 (46)	46 (41.1)	11 (9.9)	10 (8.9)
Ambrosy (2020) [18]	275 (92)	254 (91)	N/A	N/A	119 (40)	122 (44)	N/A	N/A	N/A	N/A	N/A	N/A

**Table 4:** Pre-trial medication history of the patients in included studies. ACEI/ARB: Angiotensin-converting enzyme inhibitor/Angiotensin receptor blocker, MRA: Mineralocorticoid receptor antagonist, ARNI: Angiotensin receptor-neprilysin inhibitor, N/A: Not available

Study and year	ACEi/ARB No. (%)		Diuretic No. (%)		Beta-blocker No. (%)		MRA No. (%)		Digitalis No. (%)	
	ARNI	control	ARNI	control	ARNI	control	ARNI	control	ARNI	control
McMurray (2014) [10]	4195 (100)	4229 (100)	3363 (80.3)	3375 (80.1)	3899 (93.1)	3912 (92.9)	2271 (54.2)	2400 (57.0)	1223 (29.2)	1316 (31.2)
Velazquez (2019) [11]	208 (47.3)	214 (48.5)	262 (59.5)	240 (54.4)	262 (59.5)	263 (59.6)	48 (10.9)	40 (9.1)	41 (9.3)	35 (7.9)
Desai (2019) [12]	187 (81)	204 (88)	130 (56)	128 (55)	196 (85)	204 (88)	57 (25)	58 (25)	N/A	N/A
Solomon (2019) [13]	2074 (86.2)	2065 (86.4)	2294 (95.3)	2291 (95.9)	1922 (79.9)	1899 (79.5)	592 (24.6)	647 (27.1)	N/A	N/A
Solomon (2012) [14]	139 (93)	141 (93)	149 (100)	152 (100)	117 (79)	121 (80)	28 (19)	35 (23)	N/A	N/A
Douglas (2022) [15]	N/A	N/A	157 (94)	155 (92)	122 (73)	140 (83)	103 (62)	87 (52)	37 (22)	30 (18)
Piepoli (2021) [16]	302 (97.7)	301 (97.1)	240 (77.7)	234 (75.5)	280 (90.6)	287 (92.6)	199 (64.4)	215 (69.4)	N/A	N/A
Hiroyuki (2021) [17]	111 (100)	112 (100)	91 (82.0)	95 (84.8)	105 (94.6)	108 (96.4)	64 (57.7)	69 (61.6)	N/A	N/A
Ambrosy (2020) [18]	180 (60)	178 (64)	236 (79)	210 (76)	234 (79)	225 (81)	45 (15)	36 (13)	39 (13)	33 (12)

Quality Assessment and Publication Bias

**Figure 2:**
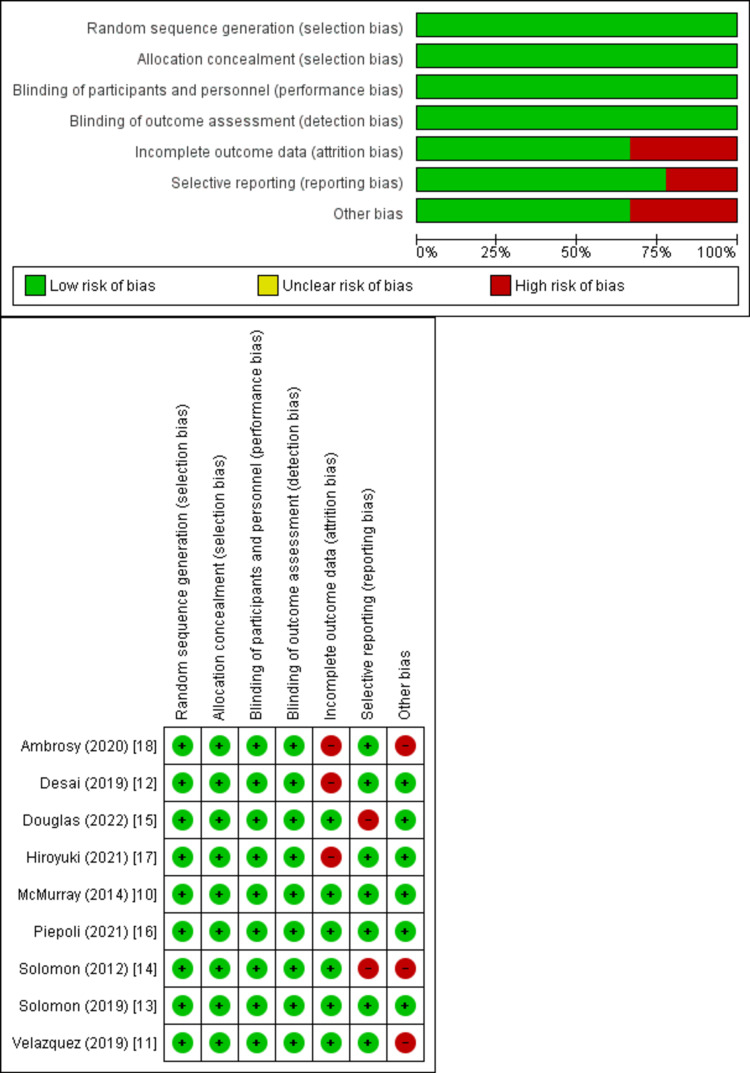
Quality assessment of the included randomized controlled trials. Source: References [10-18].

**Figure 3:**
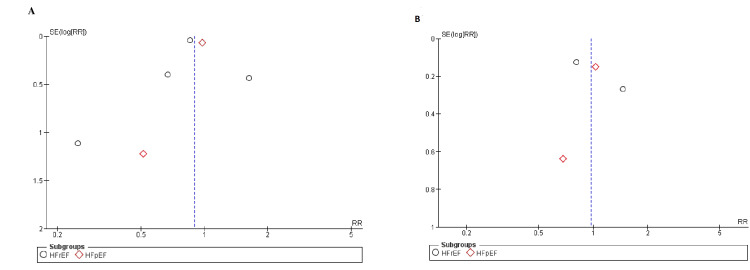
Funnel plots for (A) all-cause mortality, (B) congestive heart failure. SE: standard error, RR: relative risk.

**Figure 4:**
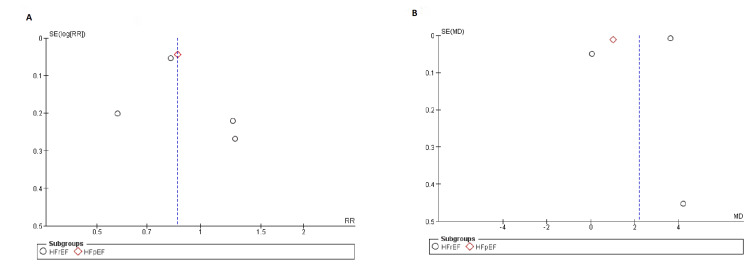
Funnel plots for (A) death from cardiovascular causes, (B) change in Kansas City cardiomyopathy questionnaire (KCCQ) clinical score. SE: standard error, RR: relative risk.

**Figure 5:**
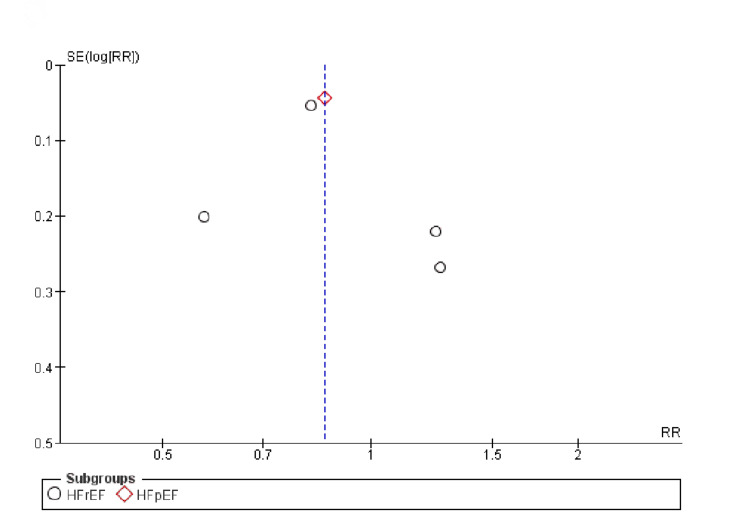
Funnel plot for first hospitalization for heart failure. SE: standard error, RR: relative risk.

The trials of medium to high quality were discovered using the Cochrane method of evaluating RCTs, as shown in Figure 2. The funnel plots showed that publication bias had no effect on the results, as shown in Figures 3-5.

Primary Outcomes

The primary outcomes analyzed were all-cause mortality, death from CV causes, first hospitalization for HF, congestive HF, and change in KCCQ clinical score.

**Figure 6:**
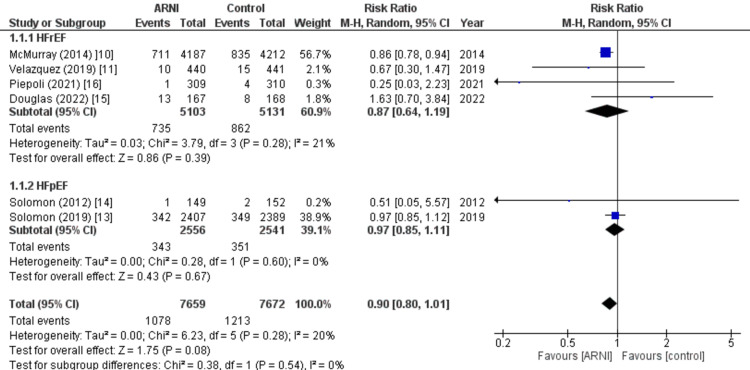
Forest plot of All-cause mortality. The figure displays the non-significant results of the pooled analysis comparing the effects of sacubitril/valsartan combination treatment to the control group on all-cause mortality risk in heart failure patients. The analysis shows a trend towards a decreased risk of all-cause mortality with sacubitril/valsartan treatment, particularly in patients with a reduced ejection fraction. RR: Relative risk; CI: Confidence interval; M-H: Mantel Hansel. Source: References [10,11,13-16].

All-cause mortality: Six of the nine studies included in the analysis reported data on all-cause mortality. The pooled analysis indicated that treatment with the sacubitril/valsartan combination was associated with a reduced risk of mortality compared to the control group, although the results were not statistically significant (RR: 0.90; 95% CI: 0.80, 1.01, p: 0.08; I2: 20%). This association was mainly observed in patients with reduced ejection fraction (RR: 0.87; 95% CI: 0.64, 1.19, p: 0.39; I2: 21%). However, it should be noted that the results mentioned above did not reach statistical significance, as indicated in Figure 6.

**Figure 7:**
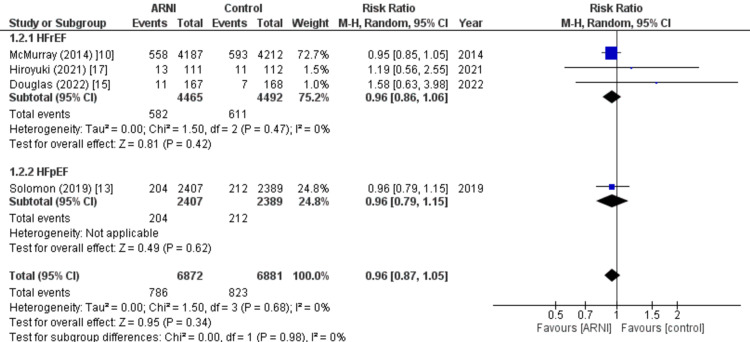
Forest plot of death from cardiovascular causes. Figure illustrating the results of the pooled analysis, which showed no significant difference in death from cardiovascular causes between the two groups across four out of nine studies, regardless of ejection fraction. RR: Relative risk; CI: Confidence interval; M-H: Mantel Hansel. Source: References [10,13,15,17].

Death from CV causes: Four of the nine studies included in the analysis reported data on death from CV causes. The pooled analysis indicated that there was no significant difference between the two groups, regardless of the ejection fraction, in terms of death from CV causes (RR: 0.96; 95% CI: 0.87, 1.05, p: 0.34; I2: 0%), as illustrated in Figure 7.

**Figure 8:**
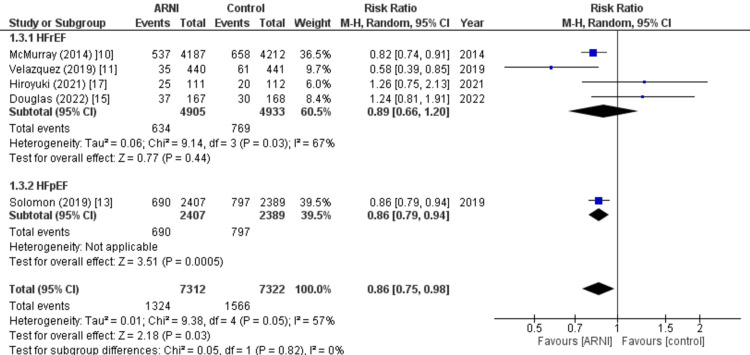
Forest plot of the first hospitalization for heart failure. Figure demonstrating the pooled analysis results revealed a significantly lower rate of first hospitalization for heart failure in the sacubitril valsartan group compared to the control group across four out of nine studies. Subgroup analysis showed this effect was particularly significant in patients with a preserved ejection fraction. RR: Relative risk; CI: Confidence interval; M-H: Mantel Hansel. Source: References [10,11,13,15,17].

First hospitalization for HF: Four of the nine studies included in the analysis reported data on the rate of first hospitalization for HF. The pooled analysis revealed that the rate of first hospitalization for HF was significantly lower in patients treated with the sacubitril/valsartan group compared to the control group (RR: 0.86; 95% CI: 0.79, 0.98, p: 0.03; I2: 57%). This effect was mainly observed in patients with preserved ejection fraction (RR: 0.86; 95% CI: 0.75, 0.94, p: 0.0005), as demonstrated in Figure 8.

**Figure 9:**
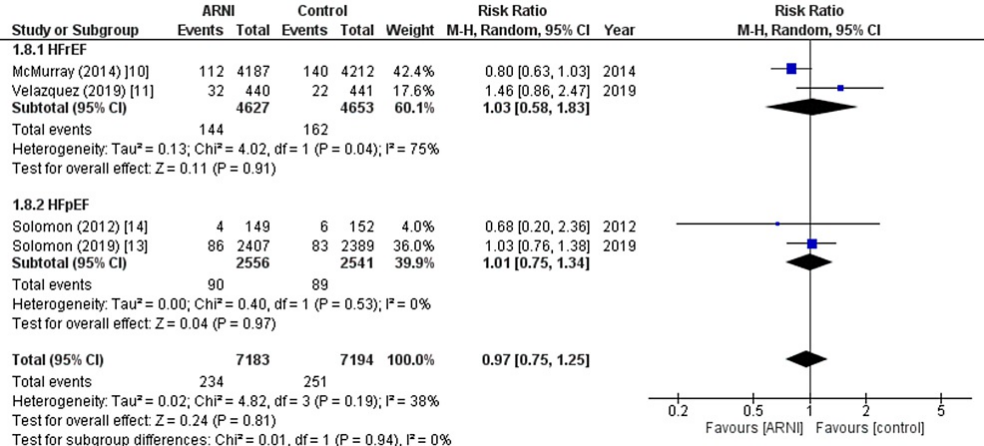
Forest plot of congestive heart failure. Figure displaying the results of the pooled analysis, which demonstrated no significant difference in the number of patients with congestive heart failure following treatment between the sacubitril valsartan group and control group across four out of nine studies, irrespective of ejection fraction. RR: Relative risk; CI: Confidence interval; M-H: Mantel Hansel Source: References [10,11,13,14].

Congestive HF: Among the nine studies included in the analysis, four of them reported data on the occurrence of congestive HF following treatment. The pooled analysis indicated no significant difference between the two groups (RR: 0.97; 95% CI: 0.75, 1.25, p: 0.19; I2: 38%), irrespective of the ejection fraction, as shown in Figure 9.

**Figure 10:**
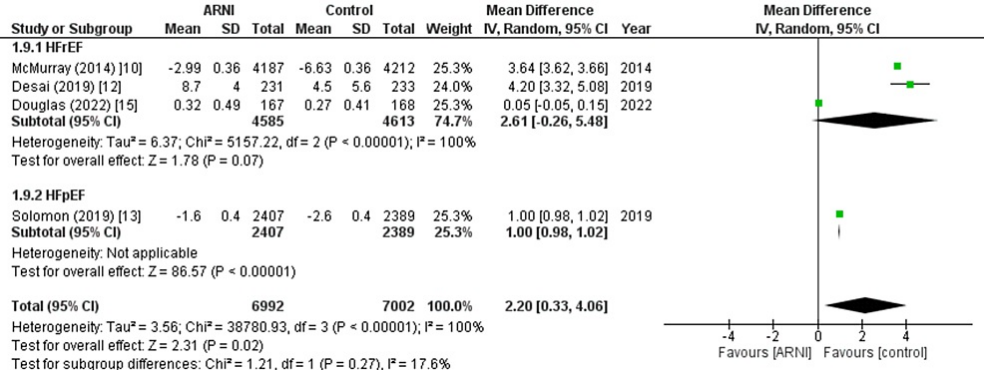
Forest plot of change in Kansas City cardiomyopathy questionnaire (KCCQ) clinical score. Figure presenting the results of the pooled analysis, which showed a significantly higher KCCQ clinical score following treatment with the sacubitril/valsartan combination compared to the control group across four studies WMD: weighted mean difference; CI: Confidence interval; M-H: Mantel Hansel Source: References [10,12,13,15].

Change in KCCQ clinical score: Four studies provided data on the change in KCCQ clinical score following treatment. The pooled analysis demonstrated that the use of the sacubitril/valsartan combination was associated with a significantly higher KCCQ clinical score compared to the control group (WMD: 2.20; 95% CI: 0.33, 4.06, p: 0.02; I2: 100%), as depicted in Figure 10. Due to the high heterogeneity observed among the included studies, a sensitivity analysis was conducted by removing one study at a time. It was found that excluding the study conducted by Desai et al. [12] did not substantially reduce the heterogeneity but rendered the results non-significant (WMD: 1.56; 95% CI: -0.57, 3.70, p: 0.15; I2: 100%).

Secondary Outcomes

The analysis of secondary outcomes in this study focused on the rate of renal function decline, hyperkalemia, symptomatic hypotension, angioedema, and TEAEs. The results of the analysis are presented in Table 5. The pooled analysis of secondary outcomes indicated that patients with HFpEF who received treatment with sacubitril/valsartan exhibited a significantly reduced risk of renal function decline. Additionally, regardless of ejection fraction, treatment with sacubitril and valsartan was associated with a significantly reduced risk of TEAEs. However, sacubitril/valsartan significantly increased the risk of symptomatic hypotension in all HF patients and angioedema in patients with preserved ejection fraction. Nonetheless, the combination therapy was not significantly associated with an increased risk of hyperkalemia in patients with preserved and reduced ejection fraction. Considering the high heterogeneity among the included studies, a sensitivity analysis excluded one study at a time. The analysis revealed that removing the study conducted by McMurray et al. [10] rendered the results non-significant (RR: 2.03; 95% CI: 0.86, 4.78, p: 0.10; I2: 96%). Conversely, removing the study conducted by Solomon et al. [13] published in 2018 reduced the in-study heterogeneity (RR: 1.37; 95% CI: 1.08, 1.74, p: 0.010; I2: 60%).

**Table 5:** Secondary outcomes. RR: Relative risk, WMD: weighted mean difference, CI: confidence interval, HFrEF: heart failure with reduced ejection fraction, HFpEF: Heart failure with preserved ejection fraction, TEAEs: Treatment-emergent adverse events.

Outcomes	Effect size (RR or WMD)	95% CI	P value	I2
Worsening renal function	RR: 0.84	0.68, 1.05	0.13	36%
HFrEF	RR: 0.93	0.79, 1.09	0.37	0%
HFpEF	RR: 0.50	0.34, 0.75	0.0007	0%
Hyperkalemia	RR: 1.07	0.91, 1.25	0.43	50%
HFrEF	RR: 1.18	0.93, 1.49	0.16	54%
HFpEF	RR: 0.89	0.72, 1.10	0.30	7%
Symptomatic hypotension	RR: 1.95	1.09, 3.50	0.02	95%
HFrEF	RR: 1.43	1.10, 1.86	0.008	62%
HFpEF	RR: 4.01	0.26, 62.68	0.32	99%
Angioedema	RR: 1.63	0.71, 3.75	0.25	35%
HFrEF	RR: 1.04	0.31, 3.53	0.95	46%
HFpEF	RR: 3.43	1.20, 9.77	0.02	0%
Any TEAEs	RR: 0.84	0.74, 0.96	0.008	55%
HFrEF	RR: 0.84	0.67, 1.06	0.15	62%
HFpEF	RR: 0.88	0.76, 1.03	0.11	-

Discussion

Despite therapeutic advancements in the past three decades, HF remains a significant cause of morbidity [19]. The conventional method of treating CHF with reduced left ventricular ejection fraction (HFrEF) entails administering angiotensin-converting enzyme (ACE) inhibitors [20]. However, when ACE inhibitors are not recommended, ARBs can be a feasible substitute. Along with beta-adrenergic receptor blockers, mineralocorticoid receptor antagonists are part of the standard triple therapy. Recently, a new class of medications, ARNI, has emerged as a viable replacement for ACE inhibitors or ARBs [20]. The European Society of Cardiology (ESC) issued updated guidelines in 2016 for managing CHF, in which sacubitril/valsartan was recommended as a treatment option. Evidence from real-world data shows that the positive outcomes of RCTs are also reflected in clinical practice [21]. In the PARADIGM-HF clinical trial, it was shown that sacubitril-valsartan was able to reduce the occurrence of CV deaths or hospitalization resulting from the worsening of CHF by 20% compared to the ACE inhibitor, enalapril. Furthermore, sacubitril-valsartan's effectiveness was more significant than enalapril's in reducing all-cause mortality by 16% and impeding the progression of CHF [22].

This meta-analysis of nine RCTs with 16,637 participants compares the efficacy and safety profile of recently introduced sacubitril/valsartan to that of enalapril or valsartan. The main objectives of our investigation encompassed several endpoints, namely all-cause mortality, mortality resulting from CV causes, initial hospitalization due to HF, congestive HF, and alterations in KCCQ clinical score. Furthermore, our study evaluated several secondary endpoints, such as the rate of decline in renal function, hyperkalemia, symptomatic hypotension, angioedema, and the frequency of TEAEs. In terms of primary endpoints, the combination of sacubitril/valsartan did not result in a reduction of all-cause mortality risk when compared to the control group. Nevertheless, certain investigations have yielded varying results. A previous meta-analysis by Charuel et al. [12] found that the overall death rate for all causes among individuals with HF and reduced ejection fraction were 16% for those receiving sacubitril/valsartan and 18% for those receiving a placebo, corresponding to a RR of 0.85 (0.78). For instance, Cosentino et al. [23] documented a decline of 20% in CV mortality, and a decrease in the frequency of hospitalizations for HF was noted in the active arm (RR 0.80; 95% CI, 0.71-0.89; p < 0.001) in comparison to the standard of care (enalapril) arm. Kaplinsky et al. [24] conducted a study that demonstrated a correlation between the decrease in left ventricular ejection fraction (LVEF) and an increased risk of CV death or HF (HF) hospitalization.

Specifically, the study found that for every five-point reduction in LVEF, there was a 9% increase in the risk of CV death or HF hospitalization. This finding contrasts with the results of our study. In patients managed with sacubitril valsartan, the initial hospitalization for HF was significantly reduced compared to the control group. This finding is supported by previous research and is evident when considering efficacy results. For instance, Chen et al.'s study demonstrated that the PIONEER-HF trial's lower rates of death and rehospitalization for HF are consistent with the findings of this analysis. However, it is noteworthy that 13% of our patients experienced acute MI during the index hospitalization, 13% had moderate or severe mitral regurgitation, and 15.6% underwent percutaneous coronary intervention (PCI). Patients with these characteristics were excluded from the PIONEER-HF trial [25]. The combined analysis results revealed no significant difference in the incidence of congestive HF between the two cohorts. While LCZ696 has obtained approval from the United States Food and Drug Administration for managing HF, it has not been authorized for clinical use in Canada [26]. Nevertheless, the Canadian HF guidelines recommend replacing ACEI or ARB with LCZ696 for patients with mild to moderate HF, an ejection fraction below 40%, an elevated level of natriuretic peptide, or a history of hospitalization for HF within the last 12 months [26]. The sacubitril/valsartan combination administration resulted in a statistically significant increase in the KCCQ clinical score. Pina et al.'s study demonstrated that the initiation of sacubitril/valsartan therapy led to a rapid improvement in KCCQ scores that persisted for 12 months. A similar study found a negative association between the baseline values of NT-proBNP and KCCQ-23 OS scores (r2=0.10; p=0.009). However, a more robust negative correlation was observed between the rate of change in KCCQ-23 OS scores and NT-proBNP (r2=0.544; p<0.001) [26]. In previous meta-analyses, statistical analysis revealed a greater risk of symptomatic hypotension and angioedema in patients with HFrEF [12]. However, the absolute figures for angioedema were comparable, and the quality of evidence for these outcomes was poor. There was no significant difference in this population's likelihood of hyperkalemia or declining renal function [12]. Given heterogeneity, we discovered that the change in KCCQ clinical score had significant heterogeneity. To address this issue, a leave-one-out sensitivity analysis was carried out. It was shown that leaving out the Desai et al. [12] study did not considerably reduce the heterogeneity but did render the findings insignificant.

Our meta-analysis offers several advantages. Firstly, it has a larger sample size than previous meta-analyses since we included four additional studies, enhancing our conclusions' validity. Secondly, we utilized various plots and tests, including the funnel plot, to evaluate publication biases, and found none. Thirdly, we conducted a sensitivity analysis to assess the impact of diverse research on the pooled estimate. Furthermore, due to recent literature, our study includes two additional outcomes: the number of TEAEs and the change in the KCCQ clinical score. Finally, we performed a subgroup analysis considering both HFrEF and HFpEF, which provided a significant comparative profile of the effectiveness of sacubitril/valsartan in patients with HF. The study in question presented some limitations. Firstly, there were differences in the control arm across the trials analyzed, with six studies using enalapril and six using valsartan. This, along with variations in sample size, ethnicity, and potential disparities in baseline characteristics of the participants, could have contributed to clinical heterogeneity. The second limitation of the study pertains to the follow-up periods, which were inconsistent across the various studies included. While some studies reported more extended follow-up periods, others had shorter ones, which might have impacted the study's findings. During the quality assessment using the Cochrane risk of bias tool, specific RCTs included in the study reported instances of attrition bias and other types of biases. Therefore, to fully address this issue, additional trials are necessary to be conducted.

## Conclusions

Our meta-analysis presents clinical data that allows physicians to discuss the risk-benefits of prescribing sacubitril/valsartan to their patients with HF. This approach promotes a patient-centered methodology in a shared process of medical decision-making. Our research demonstrates that the utilization of sacubitril/valsartan (LCZ696) is associated with decreased incidence of initial hospitalization for HF and an elevated KCCQ clinical score. This provides a significant advantage in the treatment of HF patients. Additionally, this treatment diminishes the possibility of deterioration in renal function and adverse events that arise from enalapril or valsartan. Furthermore, more standard quality RCTs with larger sample sizes are necessary to examine the other impacts of this therapy in individuals with HF.

## References

[1] Savarese G, Becher PM, Lund LH, Seferovic P, Rosano GM, Coats AJ (2023). Global burden of heart failure: a comprehensive and updated review of epidemiology. Cardiovasc Res.

[2] Becher PM, Lund LH, Coats AJ, Savarese G (2022). An update on global epidemiology in heart failure. Eur Heart J.

[3] Zhang Y, Yuan M, Suo Y (2022). Angiotensin receptor-neprilysin inhibitor attenuates cardiac hypertrophy and improves diastolic dysfunction in a mouse model of heart failure with preserved ejection fraction. Clin Exp Pharmacol Physiol.

[4] Jia R, Ji Y, Sun D (2022). Progress and prospects of Sacubitril/Valsartan: based on heart failure with preserved ejection fraction. Biomed Pharmacother.

[5] Jia R, Zhang X, Xu Y (2022). Effect of sacubitril/valsartan on renal function in patients with chronic kidney disease and heart failure with preserved ejection fraction: a real-world 12-week study. Eur J Pharmacol.

[6] Liu X, Liu H, Wang L, Zhang L, Xu Q (2022). Role of sacubitril-valsartan in the prevention of atrial fibrillation occurrence in patients with heart failure: a systematic review and meta-analysis of randomized controlled trials. PLoS One.

[7] Page MJ, McKenzie JE, Bossuyt PM (2021). The PRISMA 2020 statement: an updated guideline for reporting systematic reviews. BMJ.

[8] Higgins JP, Altman DG, Gøtzsche PC (2011). The Cochrane Collaboration's tool for assessing risk of bias in randomised trials. BMJ.

[9] Higgins JP, Thompson SG, Deeks JJ, Altman DG (2003). Measuring inconsistency in meta-analyses. BMJ.

[10] McMurray JJ, Packer M, Desai AS (2014). Angiotensin-neprilysin inhibition versus enalapril in heart failure. N Engl J Med.

[11] Velazquez EJ, Morrow DA, DeVore AD (2019). Angiotensin-neprilysin inhibition in acute decompensated heart failure. N Engl J Med.

[12] Charuel E, Menini T, Bedhomme S (2021). Benefits and adverse effects of sacubitril/valsartan in patients with chronic heart failure: a systematic review and meta-analysis. Pharmacol Res Perspect.

[13] Solomon SD, McMurray JJ, Anand IS (2019). Angiotensin-neprilysin inhibition in heart failure with preserved ejection fraction. N Engl J Med.

[14] Solomon SD, Zile M, Pieske B (2012). The angiotensin receptor neprilysin inhibitor LCZ696 in heart failure with preserved ejection fraction: a phase 2 double-blind randomised controlled trial. Lancet.

[15] Mann DL, Givertz MM, Vader JM (2022). Effect of treatment with Sacubitril/Valsartan in patients with advanced heart failure and reduced ejection fraction. JAMA Cardiol.

[16] Piepoli MF, Hussain RI, Comin-Colet J, Dosantos R, Ferber P, Jaarsma T, Edelmann F (2021). OUTSTEP-HF: randomised controlled trial comparing short-term effects of sacubitril/valsartan versus enalapril on daily physical activity in patients with chronic heart failure with reduced ejection fraction. Eur J Heart Fail.

[17] Tsutsui H, Momomura SI, Saito Y (2021). Efficacy and safety of sacubitril/valsartan in Japanese patients with chronic heart failure and reduced ejection fraction - results from the parallel-HF study. Circ J.

[18] Ambrosy AP, Braunwald E, Morrow DA (2020). Angiotensin receptor-neprilysin inhibition based on history of heart failure and use of renin-angiotensin dystem Antagonists. J Am Coll Cardiol.

[19] Gaziano TA, Fonarow GC, Velazquez EJ, Morrow DA, Braunwald E, Solomon SD (2020). Cost-effectiveness of sacubitril-valsartan in hospitalized patients who have heart failure with reduced ejection fraction. JAMA Cardiol.

[20] Pellicori P, Urbinati A, Shah P (2017). What proportion of patients with chronic heart failure are eligible for sacubitril-valsartan?. Eur J Heart Fail.

[21] Correale M, Mallardi A, Mazzeo P (2020). Sacubitril/valsartan improves right ventricular function in a real-life population of patients with chronic heart failure: the Daunia Heart Failure Registry. Int J Cardiol Heart Vasc.

[22] Jaffuel D, Molinari N, Berdague P (2018). Impact of sacubitril-valsartan combination in patients with chronic heart failure and sleep apnoea syndrome: the ENTRESTO-SAS study design. ESC Heart Fail.

[23] Cosentino ER, Degli Esposti D, Miceli R (2019). Sacubitril/valsartan improves both functional and echocardiographic parameters in patients with chronic heart failure with reduced ejection fraction. Curr Med Res Opin.

[24] Kaplinsky E (2016). Sacubitril/valsartan in heart failure: latest evidence and place in therapy. Ther Adv Chronic Dis.

[25] Chen DY, Chen CC, Tseng CN (2021). Clinical outcomes of sacubitril/valsartan in patients with acute heart failure: a multi-institution study. EClinicalMedicine.

[26] Yandrapalli S, Aronow WS, Mondal P, Chabbott DR (2017). The evolution of natriuretic peptide augmentation in management of heart failure and the role of sacubitril/valsartan. Arch Med Sci.

